# The Epidemiological and Clinical Aspects of Nasal Polyps that Require Surgery

**Published:** 2012

**Authors:** Ahmad Meymane Jahromi, Ayeh Shahabi Pour

**Affiliations:** 1 *Assistant Professor of Otolaryngology. Department of Otorhinolaryngology, Imam Reza Hospital, Faculty of Medicine, Mashhad University of Medical Sciences, Mashhad, Iran.*; 2 *Otolaryngology Resident. Faculty of Medicine, Mashhad University of Medical Sciences, Mashhad, Iran.*

**Keywords:** Clinical features, Epidemiology, Nasal polyposis

## Abstract

**Introduction:**

The objective of this retrospective cross-sectional study was to obtain epidemiological data from the charts of 297 patients with nasal polyposis who were operated on in a referral hospital in Mashhad and to determine the frequency of the presenting symptoms of nasal polyps.

**Materials and Methods:**

The variables recorded included age, gender, the presence of asthma or allergic rhinitis, family history, and previous treatments. We studied the main symptoms of nasal polyposis (nasal obstruction, rhinorrhea, anosmia, headache, epistaxis, snoring, and so on), as well as ear problems and facial deformity.

**Results:**

Nasal polyposis affects men (60.3%) more frequently, at a mean age of 39.5 years. The most frequent symptom was nasal blockage (81.1 %) followed by rhinorrhea (37.7%). A total of 11.1% of the patients had a history of epistaxis. Asthma was found in 10.4% of patients with nasal polyposis and the ears were affected in 5.1% of patients. In all, 7.4% of patients had first-degree relatives who suffered from asthma or allergic rhinitis.

**Conclusion:**

This study highlights the need for large-scale epidemiologic research exploring the prevalence and incidence of nasal polyposis in Iran.

## Introduction

Nasal polyps are mucosal lesions of the nasal or paranasal sinuses that can result from a response to inflammatory or infectious stimuli. They appear as smooth, round, semi-translucent masses that are most commonly found in the middle meatus and ethmoid sinuses and affect 1% to 4% of the population. Males are affected more than females and adults more than children. If it happens in childhood, mucociliary and immunodeficiency diseases must be ruled out, for example, patients with cystic fibrosis have a prevalence of nasal polyposis between 6% and 48% (1). Patients with nasal polyposis may present clinically with complaints of nasal obstruction, congestion, hyposmia, rhinorrhea, epistaxis, postnasal drip, headaches, and snoring. Although nasal polyps more commonly appear bilaterally they can also present unilaterally. In unilateral nasal masses, benign or malignant pathologies must be considered and distinguished by nasal endoscopy, CT scan, and biopsy (1).

The etiology of nasal polyps has been the subject of research for many years. Elevated levels of histamine and IgE found around polyps, and mast cells and eosinophilia found within polyps provide evidence suggesting that inflammation is a major factor in polyp formation (2). Previous studies have also revealed a relationship between nasal polyposis, aspirin intolerance, and allergic rhinitis and asthma (4, 5). The prevalence of nasal polyposis is higher in subjects with asthma than in non-asthmatics and 16.5% of asthmatic patients over 40 years of age have been shown to have nasal polyps (3).

The management of nasal polyposis can be both medical and surgical. Topical corticosteroids are drug of choice as they reduce the size of the polyp and improve nasal breathing and prevent recurrence. In patients who do not response to medical therapy or have large-sized polyps, functional endoscopic sinus surgery (FESS) is used to perform a polypectomy (4, 5). The objective of this study was to obtain clinical data from patients with nasal polyposis who were managed surgically. 

## Materials and Methods

We reviewed the hospital charts of 297 patients with nasal polyposis (unilateral and bilateral) who were operated on between 1998 and 2002 in our referral hospital in Mashhad, Iran. The procedures performed included simple polypectomy, the Caldwell-Luc procedure, external ethmoidectomy, and functional endoscopic sinus surgery (FESS). In all cases, the patient’s medical history and notes from an otolaryngological physical examination were reviewed and a check list of 22 variables was completed. The variables included age, age of onset, gender, season of referral, location of nasal symptoms (bilateral or unilateral). History of allergic rhinitis, asthma, or cystic fibrosis was extracted from the hospital records. Symptoms of nasal polyps such as nasal obstruction, rhinorrhea, facial pain and headache, epistaxis, snoring, mouth breathing, voice changes, ear problems, and facial deformity due to polyposis were also included in the check list. Family history of asthma or allergy and previous medical or surgical treatments were other variables that were routinely present in the hospital records of our patients in the Otorhinolaryngology ward. All of our patients had a histologic diagnosis of inflammatory nasal polyps. 

For the quantitative data, descriptive statistical analysis was conducted to determine the mean and standard deviation. For the qualitative data, we calculated percentages of the recorded variable. Our study was approved by the Institutional Board Review of Mashhad University of Medical Sciences.

## Results

Among the 297 patients with nasal polyps, 118 were female (39.7%) and 179 were male (60.3%). The average age of the patients with nasal polyps included in the study was 39.49 ±16.63 years old, with a range of 7 to 79 years old. The average age of onset was 29.2 ± 15.93 years old. In respect to age and age of onset, nasal polyps were most common in the second decade of life, followed by the third and then fourth decades. Most of the referrals occurred in the spring (36.7%) and summer (23.2%). In 161 (54.2%) of the patients the polyps were bilateral. Out of the total number of patients with nasal polyps, 31 (10.4%) presented with associated asthma and received treatment, 54 (18.2%) presented with allergic rhinitis, and 22 (7.4%) indicated that they had a first-degree relative (father/mother, brother/sister, son/daughter) who suffered from asthma or allergy. None of the patients had been diagnosed with cystic fibrosis.

**Fig 1:**
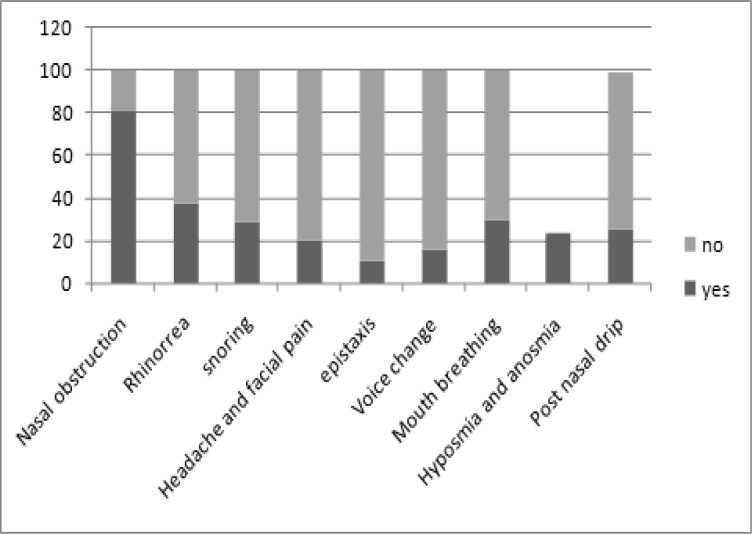
Frequency of major symptoms of nasal polyps (%)

The frequency of the major symptoms of nasal polyps in the patients studied can be seen in Figure 1. 

The most common symptom among patients was nasal obstruction (81.1%). In our study 11.1% of patients had a history of epistaxis. A total of 15 patients out of 297 (5.1%) had otorrhea, signs and symptoms of chronic otitis media, or otitis media with effusion according to history, otoscopy, and tympanometry. In 8 patients (2.7%) their nasal polyps had caused facial deformity, 141 patients (44.1%) had received medications before surgery, and 74 patients (24.9%) had a previous history of polypectomy.

## Discussion

Nasal polyposis is a condition that more commonly affects middle-aged men (6). In our study, the peak age of presentation was in the second decade of life and the mean age of patients was 39.34 ± 16.63 years. In a Nigerian district hospital, Chukuezi reported that the maximum presentation rate was between 31 and 40 years old (7). In France, the estimated incidence of nasal polyposis increased with age, reaching a peak in the 50 to 59 year age group (8). In another study in France the mean age of patients was 49.4 ± 17.6 (9). Thus, our patients were younger than in previous studies. In the only epidemiologic study of nasal polyposis in Iran that we could find, Hashemian and colleagues reported that the incidence of polyposis in 192 patients with chronic rhinosinusitis as 40%, while the sex distribution of the patients with polyposis was 60% male and 40% female and 43% of the patients also had a history of allergy (10). 

Patients with nasal polyposis often present with associated asthma. Asthmatic patients older than 40 years have a four times greater risk of suffering nasal polyposis than those under 40 years of age (3). In addition, Slavin and colleagues reported that patients with nasal polyposis present with more severe asthma than those without polyps (11). In our study, asthma was found in 10.4% of patients, a rate that was significantly lower than that found in previous studies in France (45%) and Spain (36.6%) (12, 13).

In contrast to the association with asthma, it is rare for patients with allergic rhinitis to present with nasal polyposis. Settipane and Chafee found that only 1.5% of patients with allergic rhinitis had nasal polyposis and that it was more common to find nasal polyposis in patients with non-allergic rhinitis than in those with allergic rhinitis; a difference that was statistically significant (4). 

In our sample, we found that the incidence of allergic rhinitis among patients with nasal polyposis was 18.2% in contrast to 47.9% in Spanish patients in a study by Monus (13).

In our patients, 45.8 % had unilateral nasal polyposis and it was more common in comparison with other studies such as that by Tritt and colleagues in which only 46 patients out of 301 patients with nasal polyposis had unilateral polyps and patients with unilateral polyps were younger at presentation (mean age: 35) (1). The lower incidence of asthma and allergic rhinitis and younger age of our patients could be explained by higher number of cases of unilateral polyps included in our study.

The most common complaint of patients with nasal polyposis is nasal obstruction. In our study the most frequent symptoms were nasal obstruction (81.1%) and rhinorrhea (37.7%), followed by mouth breathing and snoring. Hyposmia, headache and facial pain were less common. In our study, 11.1% of patients had a history of epistaxis although it had the lowest incidence among other symptoms but it was still high when comparing other studies. Previous studies often did not mention this symptom, probably because of low incidence (14), but Tritt and colleagues demonstrated that unilateral epistaxis in the presence of unilateral nasal polyps is statistically significant for inverted papilloma (1).

## Conclusion

An overview of the currently available literature illustrates the paucity of accurate information on the epidemiology of nasal polyposis especially in Iran, and highlights the need for large-scale epidemiologic research exploring the prevalence and incidence of nasal polyposis.
